# Calix[4]resorcinarene Amide Derivative: Thermodynamics of Cation Complexation Processes and Its Remarkable Properties for the Removal of Calcium (II) from Water

**DOI:** 10.3390/ijms26168043

**Published:** 2025-08-20

**Authors:** Angela F. Danil de Namor, Ahmad Jumaa, Nawal Al Hakawati

**Affiliations:** 1 Laboratory of Thermochemistry, School of Chemistry & Chemical Engineering, University of Surrey, Guildford, Surrey GU2 7XH, UK; 2 Department of Biological Sciences, Faculty of Science, Beirut Arab University, Tripoli P.O. Box 11-5020, Lebanon; n.elhakawati@bau.edu.lb

**Keywords:** calix[4]resorcinarene amide, calcium ion, selectivity, capacity

## Abstract

The state of the art in the thermodynamics of calix[4]resorcinarene derivatives and its metal ion complexes is briefly discussed in the introduction. This is followed by the synthesis and characterization of a recyclable calix[4]resorcinarene amide derivative (L). The 1H NMR analyses in CD3CN and CD3OD showed solvent-dependent conformational changes with a notable downfield chemical shift in the aromatic proton (H-2) in moving from deuterated methanol to acetonitrile, indicating an interaction of the solvent within the ligand cavity as suggested by molecular dynamic simulations. 1H NMR complexation in acetonitrile revealed that L forms relatively strong 1:1 complexes with cations, with selectivity for Ca(II) and, to lesser extent, with Pb(II) over other metal cations. The composition of the complexes is corroborated by conductance measurements. The thermodynamics of these systems indicate that the complexation process is predominantly enthalpy controlled in acetonitrile, while it is entropy controlled in methanol. A remarkable outcome of fundamental studies is found in its application as new material for the removal of Ca(II) from water. The capacity of **L** to remove Ca(II) from water is 24 mmol/g which exceeds by far the capacity of cation exchange resins.

## 1. Introduction

The chemistry of calix[4]resorcinarenes has been the subject of numerous papers and review articles mainly dealing with synthesis, structural characterization, thermal behaviour [1,2], capsules resulting from the formation of self-assemblies [3] and starting materials for the synthesis of cavitants [4], carceplexes, and hemicarceplexes [5]. Calix[4]resorcinarenes and their derivatives have found a wide range of applications in several areas such as iodine capture [6], sensors [7], antioxidants [8], and catalysts [9,10]. One of the main features of Supramolecular Chemistry is the ability of macrocycles to form complexes with ionic and neutral species. Within this context, the issue of selectivity for experimental applications is of outmost importance, and it is on the basis of detailed thermodynamics that a quantitative assessment can be made on the selective recognition of a given receptor for one species relative to another in a given medium and temperature. However, the state of the art is such that despite the large number of papers and reviews in this area, thermodynamic studies on calix[4]resorcinarene and its derivatives and guest species are limited and often not representative of the process taking place in solution. Such is the case for data reported for complexation studies involving calix[4]resorcinarene derivatives [esters and amides] and cations in deuterated chloroform [11]. In this medium, predominantly ion pairs are present in solution and therefore the data are not referred to a defined process as previously stated in the literature [12]. Thermodynamic aspects of complexation of mainly organic cations and a calix[4]resorcinarene in water-organic media have been investigated by Mustafina and co-workers [13], while those involving soft metal cations and partially and fully substituted calix[4]resorcinarenes have been reported by Danil de Namor and co-workers [14,15,16]. As far as thermodynamic studies of the receptors are concerned, these have been carried out on calix[4]resorcinarene derivatives in water and nonaqueous solvents by Riveros and co-workers [17,18,19]. Enthalpies of solution of these receptors in water derived from solubility measurements of the appropriate receptor in water at various temperatures by the use of the van’t Hoff equation are only approximate values due to the well-known limitations of this equation for the calculation of enthalpy values [12].

It is important to emphasize that in our work, the term thermodynamics embraces not only the complexation process (Equation (1)) but also the solution processes of the reactants and the product participating in the complexation process (Equations (2)–(4)) in a given solvent. When two solvents are involved, the thermodynamic parameters of transfer from a reference solvent (s_1_) to another (s_2_) are calculated (Equations (5)–(7)). Given that in the transfer parameter, the crystal lattice energy is eliminated, the values obtained reflect the differences in solvation of reactants and product from one medium to another. Each of these processes are characterized by corresponding values of Gibbs energy, ΔG^0^, enthalpy, ΔH^0^, and entropy, ΔS^0^ referred to a defined standard state (1 mol.dm^−3^).

M^+^(s) + L (s) → M^+^L (s) (1)

MX (solid) → M^+^ (s) + X^−^ (s) (2)

L (solid) → L(s) (3)

MLX (solid) → M^+^L (s) + X^−^ (s) (4)

M^+^ (s_1_) + X^−^ (s_1_) → M^+^ (s_2_) + X^−^ (s_2_) (5)

L (s_1_) → L (s_2_) (6)

M^+^L (s_1_) + X^−^ (s_1_) → M^+^L (s_2_) + X^−^ (s_2_) (7)

In the general field of Supramolecular Chemistry, thermodynamic data have not only been able to give a quantitative measure of the selective behaviour of a receptor for a substrate relative to another in a given medium but have led to important developments as demonstrated in the following examples.

(i) As far as the cryptands are concerned, the realization that in the complexation of these receptors with univalent cations in dipolar aprotic solvents, the cations entirely lose their solvation shell when entering the cavity of the receptor led to a new extra-thermodynamic convention for the calculation of single-ion transfer data for anions among dipolar aprotic solvents [20,21,22].

(ii) Thermodynamics revealed that electrolytes containing the lithium coronand cation are less solvated than those containing the bare cation. These findings were corroborated by the considerable increase in conductivity of the former relative to the latter and their potential use in lithium battery technology [23,24].

(iii) The additive thermodynamic contribution of pendant arms to the complexation of calix[4]arene derivatives with mercury (II) [25].

(iv) Complexation thermodynamics involving calix[4]-based derivatives and cations in acetonitrile were shown to be a suitable reporter of selectivity coefficients in ion-selective electrodes based on calix[4] receptors [26,27].

The aim of this paper is to explore whether or not some of the fundamental aspects of the complexation reactions involving calix[4]arene amide derivatives and metal cations in a dipolar aprotic solvent (acetonitrile) and a protic solvent (methanol) are observed for the resorcinarene-based receptor given that its synthesis is considered to be simpler than that of calix[4]arenes and the number of functional groups in the former is twice that in the latter. Therefore, we report the following here:

1. Synthesis and structural characterization of a new calix[4]resorcinarene derivative, **L** containing amide pendant arms.
2. ^1^H NMR studies on the interaction of **L** with metal cations in CD_3_CN and CD_3_OD. These are limited to assess whether the receptor interacts with the cations and, if possible, to identify the sites of ligand–metal cation interaction. For this work, these measurements are useful to avoid proceeding with detailed thermodynamic studies for those cations for which the NMR data shows no interaction with the ligand in the appropriate solvent.
3. Conductometric studies to determine the composition of the complex in acetonitrile and methanol.
4. A detailed thermodynamic study for the complexation of **L** and metal cations in acetonitrile and methanol to obtain quantitative information on the stability of the complexes and therefore the selectivity of **L** for a given cation relative to others. This information is of utmost importance for the selection of the receptor in the removal of cations from water.
5. Extraction of Ca(II) from water by **L** under optimal experimental conditions.

## 2. Results and Discussion

### 2.1. ^1^H NMR Characterization of ***L*** in Different Deuterated Solvents at 298 K

L was further characterized by ^1^H NMR in two nonaqueous deuterated solvents namely, acetonitrile, CD_3_CN (dipolar aprotic), and methanol, CD_3_OD (protophilic). The chemical shift values (δ, ppm) and the chemical shift changes (Δδ, ppm) of **L** relative to CD_3_OD (reference solvent) are listed in Table 1. By analyzing these shifts, it is possible to gain insight into how **L** interacts with the solvent environment and whether any conformational changes occur.

Inspection of chemical shifts for L in these two deuterated solvents reveals different behaviour of the ligand in these media. It is observed that in deuterated acetonitrile, downfield chemical shift change is observed mostly in H-2 proton relative to its position in CD_3_OD. This statement is corroborated by a slight upfield shift in the CH_3_ (H-7) of the acetamide functionality of the receptor. This shift must be attributed to changes in the ligand’s conformation, indicating an interaction between CD_3_CN and the ligand’s cavity as previously demonstrated for other calix[4]arene derivatives [12]. As a result, the electronic environment of nearby protons is altered. Interestingly, other protons of L specifically H-3, H-4, H-5, and H-6 do not exhibit any significant chemical shift changes in moving from one solvent to another. This suggests that these regions of the ligand experience little or no perturbation from the solvent, reinforcing the fact that the primary structural or electronic effects of the solvent are localized around the H-2 and to a much lesser extent to the acetamide moiety (H-7).

**Figure 1:**
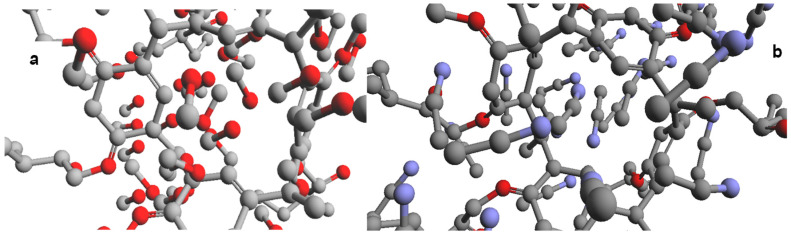
Molecular simulations of L in methanol (**a**) and acetonitrile (**b**) using molecular mechanics with the universal force field (UFF). Structures represent energy minimized conformations showing the behaviour of **L** in the solvents.

Molecular dynamic simulation studies were carried out with the aim of providing suggestions regarding the solvation dynamics, hydrogen bonding, solute–solvent interactions, and the stability of solute conformations in different solvents. Methanol (MeOH) is a polar protic solvent capable of forming strong hydrogen bonds with the hydroxyl (-OH) and acetamide (-C(O)NH_2_) groups of calix[4]resorcinarene. Simulations show that methanol molecules (Figure 1a) form a solvation shell around the resorcinarene macrocycle, stabilizing it through H-bonding. Due to its polarity and protic nature, methanol provides a high solvation and stabilizes the molecule in a relatively open conformation. As for acetonitrile (MeCN), it is a dipolar aprotic solvent with a high dipole moment but lacks hydrogen bonding capability. The acetamide group (-C(O)NH_2_) may engage in dipole–dipole interactions with MeCN. However, compared to methanol, the absence of strong hydrogen bonding may lead to weaker solvation. The L adopts a more compact structure in MeCN (Figure 1b) than in MeOH.

### 2.2. ^1^H NMR Investigations on the Complexation of ***L*** with Metal Cations in CD_3_CN and CD_3_OD at 298 K

Table 2 shows the ^1^H NMR proton resonances (δ) and chemical shift changes with uni- and bivalent cations when changes are observed in CD_3_CN and CD_3_OD at 298 K. It is quite clear from Table 2 that as result of complexation the ligand undergoes significant conformational changes reflected in the downfield chemical shift changes observed for H-1, while upfield chemical shifts are mostly found for H-2.

A possible suggestion for alkali-metal cations (Na(I), K(I), Rb(I)) that the downfields observed for H-5 may be attributed to an interaction between the cation and the carbonyl oxygen and the nitrogen donor atoms of the ligand.

As far as the alkaline-earth metal cation are concerned, the downfield chemical shift changes observed in H-4 may result from the carbonyl oxygen providing the sites of interaction with the cation. Data for Cd(II) and Zn(II) are similar due that their similar structures and electronegativity. They are also in the same group in the Periodic Table. For these cations chemical shift changes are found in H-1, H-3, H-4, and H-5. Interaction with Pb(II) occurs through H-5, and is likely to result from the participation of both donor atoms, the carbonyl oxygen and the amide nitrogen. Finally, the downfields shown by the addition of Hg(II) to the receptor for H-1 and it can be attributed to a cation–pi noncovalent interaction. 

The pattern found for ^1^H NMR complexation studies in CD_3_OD differs significantly from those in CD_3_CN. It is not only due to the higher solvation of ions in the former solvent relative to the latter, but also to the higher solvation of L in CD_3_OD with respect to CD_3_CN, as suggested by molecular simulation studies (Figure 1). Table 2 also lists the chemical shift changes in the ^1^H NMR of **L** upon the addition of excess amounts of metal cations in CD_3_OD at 298 K. As a result of the addition of alkali-metal cations to L, no complexation was observed in methanol. Similar results are produced by the complexation of L with Mg(II), Zn(II), Cd(II), and Hg(II) in methanol. A significant change in the chemical shift in the aromatic proton (H-2) appeared after the addition of Ca(II), Sr(II), Ba(II), and Ag(I) to **L** in methanol. As far as Pb(II) is concerned the significant chemical shift change in the methylene bridge proton is possibly due to the change in conformation of the ligand upon complexation. Within the context of this work, the contribution from ^1^H NMR studies is to provide information regarding whether cation–ligand interaction takes place in order to select the cations to proceed with thermodynamic studies. Essentially it avoids proceeding with the latter studies when the ^1^H NMR spectra showed that cation–ligand interactions are non-existent. No attempts are made to propose the structure of the complexes from the NMR data. 

Conductometric titrations are used to determine the composition of the complexes between macrocycles and metal cations in solution. It also gives an indication of the strength of complexation. In this work conductometric titrations were carried out between **L** and all cations. A representative example is given for the calcium cation in both acetonitrile and methanol at 298.15 K. 

**Figure 2:**
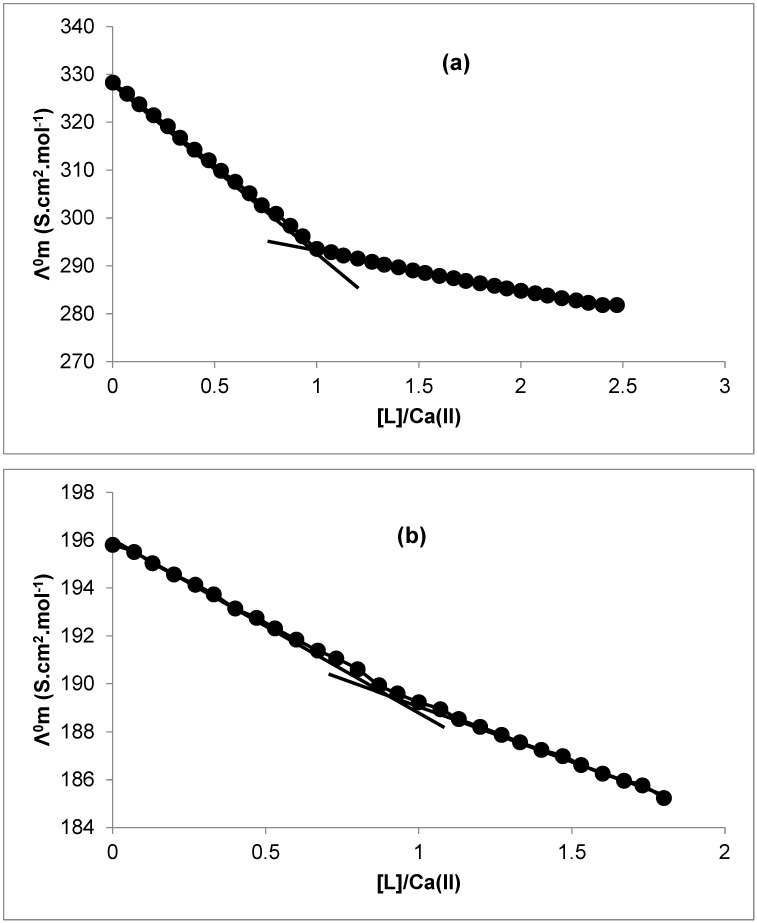
Conductometric curve for the titration of Ca(II) (perchlorate as counter-ion) with **L** in acetonitrile (**a**) and methanol (**b**) at 298.15 K.

The results of the conductometric titrations of calcium cation (perchlorate as counter-ion) with **L** in acetonitrile and methanol at 298.15 K are presented in Figure 2, thus giving a qualitative indication on the strength of complexation of the metal cation with the ligand. The following graphs are plots of molar conductance Ʌm (S.cm^2^.mol^−1^) against ligand: metal–ion concentration ratio ([**L**]/[M^n+^]) during the titration of the salt with the ligand. Figure 2 shows the conductometric titration curves for the metal cation and **L** in acetonitrile and methanol at 298.15 K. In the former solvent, the titration curve shows a decrease in the conductance until reaching a 1:1 ligand/metal–ion stoichiometric ratio. The sharp change is due to the formation of a complex between **L** and the ions in the solvent, which decreases the mobility of the cation. The intersecting lines drawn in the figure indicate that strong complexation with 1:1 (ligand/metal–ion) stoichiometry occurs. As far as the conductometric titration curve for Ca(II) and **L** in methanol is concerned (Figure 2), the result shows the formation of a weaker 1:1 complex in MeOH relative to MeCN. 

### 2.3. Thermodynamic Parameters of Complexation of L with Metal Cations in CH_3_CN and CH_3_OH at 298.15 K

^1^H NMR and conductometric studies gave qualitative results of the complexation of L with metal cations in acetonitrile and methanol. In order to quantitatively determine the stability constant (log K_s_) of the complex (hence the Gibbs energy, Δ_s_G°) as well as the contribution of the enthalpy (Δ_c_H°) and entropy (Δ_c_S°) to the stability of the complex, calorimetric titrations were carried out for the complexation of **L** and metal cations in acetonitrile and methanol. Data in both solvents are listed in Table 3**.** Data are referred to the standard state (1 mol.dm^−3^) for reactants and the product.

As shown in Table 3, the selectivity of **L** for cations in acetonitrile follows the sequence Ca(II) > Pb(II) > Ba(II) > Sr(II) > Cd(II) > Hg(II) > Mg(II) > Zn(II).

In all cases, the stability of the complex is enthalpy controlled. 

The two processes which mainly contribute to the overall complexation between a ligand and a metal cation in solution are the ligand binding energy and the cation desolvation [19]. The balance between these two processes is reflected in the stability constant. There is hardly any data on the Gibbs energies of solvation, *Δ_solv_G^0^*, of bivalent cations either in acetonitrile or methanol. Given that,

(8) $$\Delta_{solv}G^{0}\left(M^{n+}\right)={∆}_{h}G^{0}\left(M^{n+}\right)+{∆}_{t}G^{0}{\left(M^{n+}\right)}_{\left(H_{2}O\to s\right)}$$

**Figure 3:**
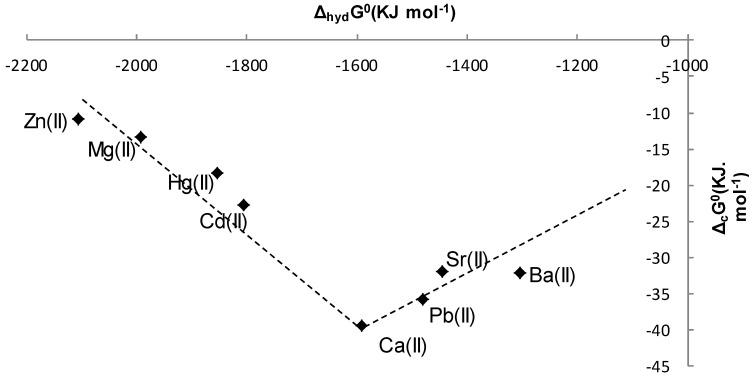
Δ*_c_G*^0^ Values for **L** against Δ*_h_G*^0^ of bivalent metal cations in acetonitrile.

Considering that the *Δ_t_G^0^* values for the transfer of these cations from water to a nonaqueous solvent are relatively small when compared with the *Δ_h_G^0^* of these cations, it follows that the trend in the *Δ_solv_G^0^* would not differ from that of the *Δ_h_G^0^*. The latter parameter is used with the aim of assessing whether or not there is a correlation between the Gibbs energies of complexation and those of hydration. This is shown in Figure 3. 

This figure shows that the two forces (binding and the desolvation) compensate showing a selectivity peak. When the absolute binding energy overcomes the increased energy required for cation desolvation, the stability of complex formation increases. Thus, a maximum stability is reached for Ca(II), after which the binding energy is not enough to overcome that required for desolvation, and therefore log Ks values become smaller. Similar results have been achieved when the *p*-tert-Butylcalix[4]arene-tetraacetone was investigated in acetonitrile with bivalent metal cations [28]. This similarity in the results and the trend gives an indication that the ligand being investigated in this work, **L**, has an active site which may be limited to the carbonyl oxygen and the ethereal oxygen. Thus, the nitrogen atom of the amide group does not seem to participate in complex formation.

**Figure 4:**
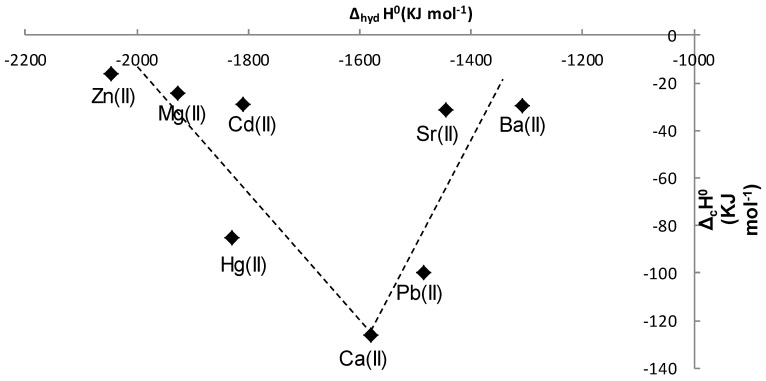
Δ*_c_H*^0^ values for **L** against Δ*_h_H*^0^ of bivalent metal cations in acetonitrile.

**Figure 5:**
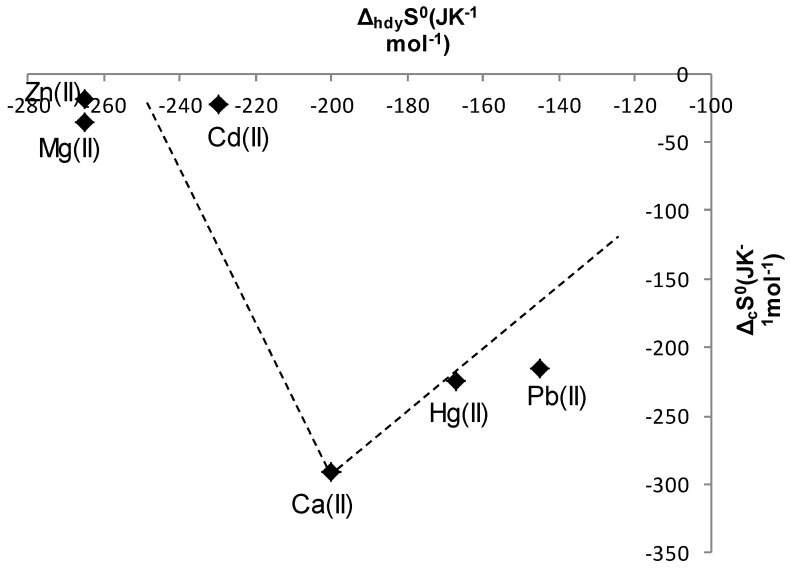
Δ*_c_S*^0^ values for **L** against Δ*_h_S*^0^ of bivalent metal cations in acetonitrile.

In order to assess whether the selectivity peak observed in terms of Gibbs energies is enthalpy or entropy controlled, the hydration enthalpies of bivalent cations in acetonitrile are considered. Hydration enthalpies [29,30] were used to establish whether a relation is found between these data and the *Δ_c_H^0^* values reported in Table 3. Thus, a plot of *Δ_c_H^0^* against *Δ_h_H^0^* is presented in Figure 4. This plot is similar to that shown in Figure 3 in that the exothermic maximum is found for Ca(II), therefore it agrees with the selectivity peak observed in terms of Gibbs energies. However, there are entropic factors that deviate from the linearity observed in terms of Δ_c_G^0^, as shown in Figure 5. 

The *Δ_c_S^0^* values being calculated from Gibbs energy and enthalpy data. In order to assess the medium effect on the complexation process, thermodynamic parameters of the complexation of **L** and metal cations in MeOH were determined at 298.15 K and are listed in Table 3. The table contains values of the stability constant (log K_s_), standard Gibbs energies (*Δ_c_G^0^*, kJ.mol^−1^), enthalpies (*Δ_c_H^0^*, kJ.mol^−1^), and entropies of complexation (*Δ_c_S^0^* J.K^−1^.mol^−1^) of **L** with metal cations in methanol at 298.15 K. Values for Ba(II) and Pb(II) are below the lower detection limit of calorimetry and therefore may be regarded as approximate log values.

The complexation reaction between L and bivalent metal cations (alkaline-earth and heavy metals) is represented in Equation (9)

(9) $$M^{2+}\left(MeOH\right)+L\to \;M^{2+}L\left(MeOH\right)$$

Considering first the alkaline-earth metals, it is clear that there is complexation between **L** and Ca(II). However, the complexation with Ba(II) is very weak. The bivalent heavy metal cations listed in the table undergo a very weak complexation with **L** in methanol at 298.15 K.

The stability constant data shown in Table 3 indicate that the interaction of **L** with metal cations in methanol is selective for Ca(II). The log K_s_ values result from different enthalpy and entropy contributions. Thus, for Ca(II), enthalpic ability is relatively low and the favourable entropy is indicative that the metal cation may undergo strong desolvation upon complexation. As a result, the process is entropy controlled. However, the interaction between **L** with either Ba(II) or Pb(II) in methanol is enthalpy controlled. The log K_s_ values follow the sequence:

Ca (II) > Pb (II) > Ba (II)

### 2.4. Calcium, Mercury, and Lead Ions Interaction with ***L*** Using Molecular Simulation

**Figure 6:**
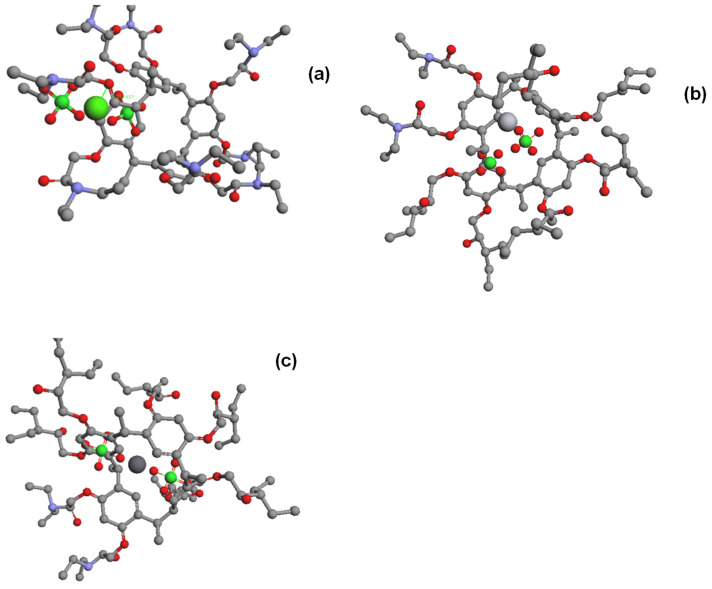
Molecular simulations showing the interaction of **L** with Ca(II) (**a**), Hg(II) (**b**), and Pb(II) (**c**) as perchlorate. Ion–dipole interactions are represented by green lines (**a**).

The interaction of the calcium, mercury, and lead ions with the **L** ligand was simulated using ArgusLab 4.0.1 software to elucidate the key forces governing their association. The objective was to gain suggestions into the nature of the intermolecular interactions between the ligand and these ions, particularly in terms of stability and binding characteristics. As shown in Figure 6, the analysis reveals a change yet consistent stability in the system, primarily attributed to ion–dipole interactions between the carbonyl (CO) functional group of **L** and the calcium ion. This interaction suggests a well-defined 1:1 binding mode, where the lone pair of electrons on the oxygen of the carbonyl group coordinates directly with the Ca(II) ion. However, for the interaction of **L** with Hg(II) and Pb(II), these cations appeared in the ligand’s cavity showing a conformational change in its aromatics and that is what was revealed from the ^1^H NMR analysis (Table 2).

From an energetic view, the binding energy values for Hg(II)-L and Pb(II)-L were −16.73 and −20.92 kJ/mol, respectively. Whereas the calcium-L interaction was predominantly enthalpically driven with a binding energy of −37.65 kJ/mol, indicating favourable electrostatic and coordination contributions to the binding stability. The enthalpic stabilization arises from the strong electrostatic attraction between the divalent calcium ion and the partial negative charge on the carbonyl oxygen, complemented by possible polarization effects that enhance the ligand’s affinity for the metal centre. Furthermore, the absence of significant steric hindrance or competing interactions reinforces the stability of the complex. These results provide useful suggestions into the fundamental forces at play and potential implications for calcium-binding process in related systems.

### 2.5. Extraction of Calcium Ion from Water Using ***L***

#### 2.5.1. Determination of Optimum Amount of **L** for the Removal of Calcium from Water at 298 K

**Figure 7:**
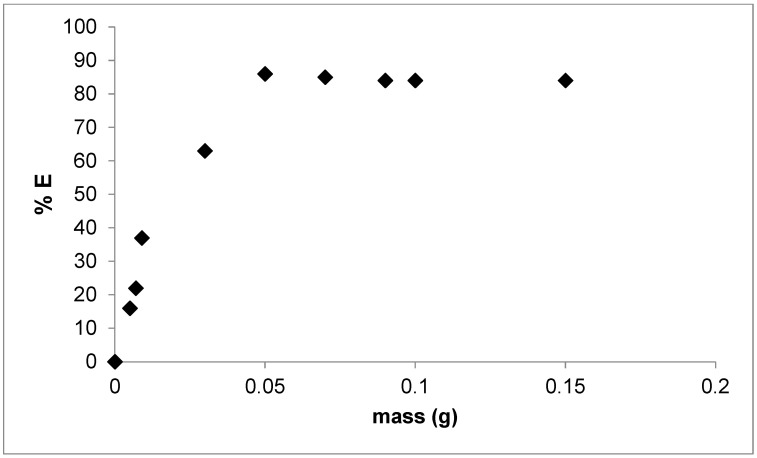
Effect of mass on the extraction of Ca(II) from aqueous solution by **L** at 298 K, C_i_ = 1 × 10^−1^ mol dm^−3^, V = 10 cm^3^, and pH~7.

To determine the optimum mass of **L** required for optimal calcium extraction from water, batch experiments were conducted using different amounts of the material. The results, presented in Figure 7, indicate that **L** was capable of removing up to 86% of calcium ions, with an optimal ligand mass of 0.05 g.

The observed increase in calcium removal with increasing L mass can be attributed to the greater availability of active binding sites. As more material is introduced into the system, the number of sites available for ion removal increases, thereby enhancing the overall uptake capacity for a given initial ion concentration. However, beyond a certain mass, the system reaches a saturation point, where further increases in L mass do not result in a significant calcium removal. This suggests that at 0.05 g, the material effectively captures the majority of available calcium ions. 

These findings highlight 0.05 g of L as the optimal dosage for calcium removal under the investigated conditions, providing an effective balance between material efficiency and extraction performance. This information is crucial for practical applications, as it allows for the design of cost-effective processes while ensuring maximum ion removal from aqueous solutions.

#### 2.5.2. The pH Effect on the Uptake of Calcium from Water by **L** at 298 K

**Figure 8:**
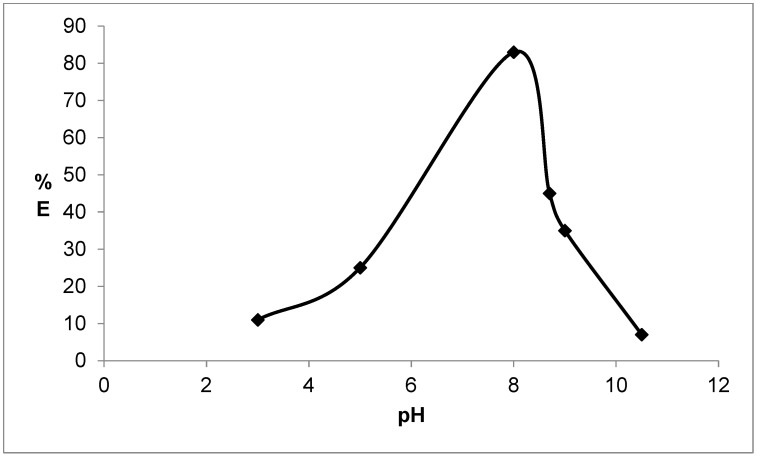
Percentage of extraction of the Ca(II) plotted against the pH using **L**, C_i_ = 1 × 10^−1^ mol dm^−3^, V = 10 cm^3^, and m = 0.05 g for **L**.

Figure 8 illustrates the effect of solution pH on the percentage of calcium ion removal by **L** at 298 K. The results indicate that calcium extraction is strongly influenced by the pH of the solution, with the highest removal observed at an optimum pH of approximately 8. At this pH, L demonstrates maximum binding affinity for calcium ions, leading to enhanced sequestration from the aqueous phase.

To interpret these findings, it is essential to consider both the dissociation behaviour of calcium species in solution and the availability of donor atoms on the L ligand that participate in calcium coordination. In aqueous media, calcium perchlorate Ca(ClO_4_)_2_ behaves as a strong electrolyte, undergoing dissociation into Ca(II) and ClO_4_^−^ ions. Unlike weak acids or bases, calcium perchlorate does not have a meaningful pKa value, as it does not establish any acid–base equilibrium in solution.

At lower pH values, an excess of H^+^ ions may compete with calcium for active sites on L, reducing removal efficiency. Conversely, at pH 8, the ligand is likely to exist in an optimal state for maximum calcium removal efficiency, making it a critical parameter for practical applications in water treatment and ion-exchange processes.

#### 2.5.3. Calcium Uptake Capacity of **L** at 298 K

**Figure 9:**
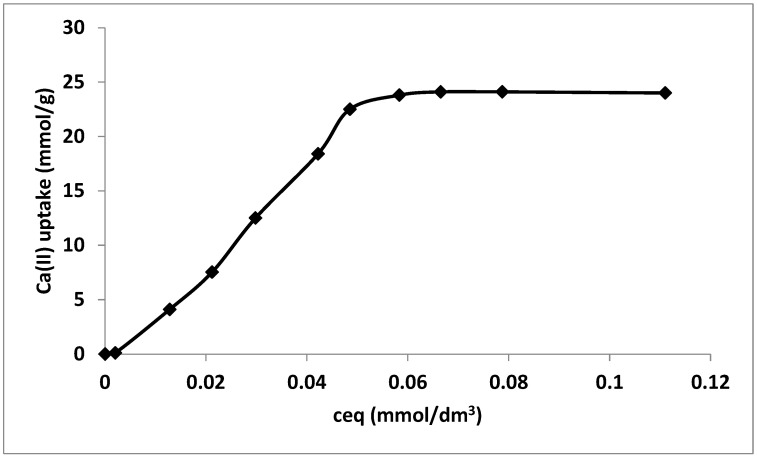
Amount of calcium (mmol/g) taken up by **L** against the equilibrium concentration of the ion in aqueous solution, V = 10 cm^3^, m = 0.05 g for **L**, and pH~8.5.

Batch experiments were conducted to evaluate the calcium uptake capacity of **L** from aqueous solutions under controlled conditions. The uptake behaviour of **L** was assessed by measuring the amount of calcium retained per gram of material as a function of the equilibrium molar concentration of calcium salt in solution. The results, presented in Figure 9, demonstrate a clear trend in calcium uptake with increasing ion concentration. Notably, **L** exhibited a high binding capacity, reaching a maximum uptake of 24 mmol of calcium per gram of material. These findings highlight the potential of **L** as an efficient material for calcium ion sequestration from aqueous environments. In fact, the uptake of Ca(II) (as perchlorate salt) by the material is due to the several individual processes taking place in the overall extracting process involving the removal of the cation salt from water to the solid phase. The capacity of the solid material is not necessarily related to the complexation process in solution, due to the fact that in the complexation process, ions are involved, but in the solid material strong association will take place with the participation of the anion to maintain the electroneutrality in the solid phase.

#### 2.5.4. Kinetics of Calcium Ion Uptake by **L** from Aqueous Solution at 298 K 

**Figure 10:**
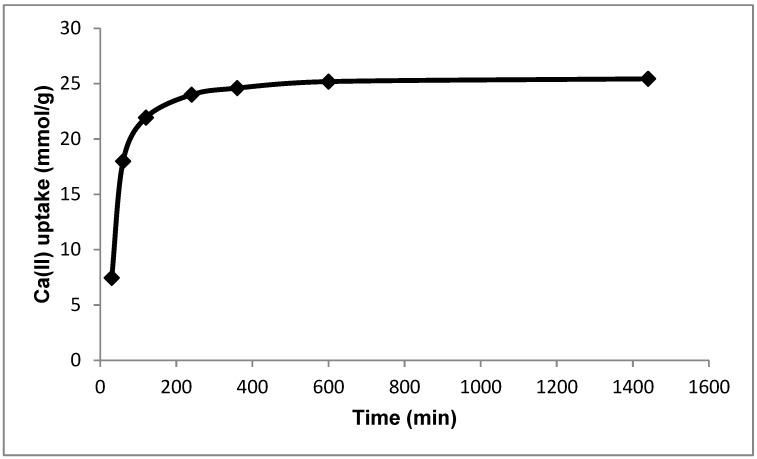
Determination of the optimum time for the uptake of Ca(II) ion **L** from aqueous solution at 298 K, V= 10 cm^3^, m= 0.05 g for **L**, pH~ 8.5. t_1/2_: 50 min. k: 0.013 min^−1^.

For commercial applications, the kinetics of the extraction process is a critical factor, as it determines the efficiency and feasibility of large-scale implementation. To assess the rate of calcium uptake by **L**, batch equilibrium experiments were conducted at 298 K while maintaining optimal conditions, including the optimal amount of **L** and a fixed solution pH. The results of these experiments, presented in Figure 10, illustrate the uptake of Ca(II)by the material as a function of contact time.

The data indicate that a significant proportion of calcium ions were removed within the first few minutes of interaction, suggesting rapid initial removal, likely due to the availability of abundant active binding sites on the **L** material. However, as the reaction progressed, the uptake rate gradually decreased, reaching the capacity of the material after approximately 4 h. Beyond this point, no appreciable changes in calcium removal were observed, indicating saturation of the available binding sites. This behaviour suggests that the process follows a two-phase kinetic model, characterized by an initial rapid removal phase followed by a slower approach to saturation. These findings demonstrate the potential of **L** for efficient calcium removal within a relatively short operational timeframe, making it a promising material for technological applications.

#### 2.5.5. Effect of Interfering Cations on Calcium Uptake from Aqueous Solution by **L** at 298 K

The ability of the investigating material to extract calcium ions from water was tested in the presence of competing metal cations, including lead (Pb(II)), cadmium (Cd(II)), barium (Ba(II)), and strontium (Sr(II)). The results confirmed that **L** maintains its selectivity for calcium ions even when these interfering cations are present in the solution. However, the presence of these additional metal ions has a noticeable impact on the overall extraction efficiency, leading to a reduction in calcium uptake by approximately 35%. This decrease in capacity suggests that competitive interactions between calcium and these other divalent cations influence the binding process. Despite this reduction, the quantity of calcium removed under the given experimental conditions remains significant, highlighting the effectiveness of **L** in calcium ion extraction even in complex aqueous environments.

### 2.6. Recycling of ***L*** Ligand for Calcium Removal via pH-Switching Mechanism

The ability to recover the **L** ligand after calcium ion removal from water is an important aspect for water treatment applications. The findings from the pH-dependent study indicate that L can highly extract Ca(II) ions at an optimal pH of approximately eight. 

The investigated pH effect provides a practical basis for regenerating the ligand through a pH-switching mechanism. Following calcium sequestration at pH 8, the ligand-loaded material was exposed to an acidic environment (e.g., pH < 2) through the use of nitric acid 0.1 M. After five regenerations the capacity of the material was reduced by 10 % which means that there is room for a much greater number of regenerations before the life cycle of the material expires.

### 2.7. Comparison with Other Materials

There are several methods for the removal of calcium from water such as precipitation, solvent extraction, and bulk liquid membranes. Among the materials, ion exchange resins are commonly used for the removal of Ca(II) from water. These resins are not selective and their capacity varies from 1 to 2.45 mmol/g [31]. Undoubtedly, selectivity of a material for a target ionic or neutral species is a key factor in extraction processes. This is clearly demonstrated in this paper where the capacity of **L** (24 mmol/g) is ten times higher than any cation exchange resin available. Even in the presence of interferences, the capacity of the material is reduced to 16 mmol/g. In addition, it is easily recyclable via a pH switching mechanism.

Most of the materials reported in the literature for the removal of cations contain calcium compounds in their structures. Thus, Chavez and co-workers [32] reported the adsorption of Ba(II) by Ca-exchange using clinoptilolite tuff and montmorillonite clay. Again, a hybrid bead composite based on silica modified calcium–alginate–xanthan gum was used for the recovery/removal of Pb(II) from acidic aqueous solutions [33]. Another contribution involves the modification of natural silica sand by calcium oxide for removing uranyl ions [34] from aqueous solutions. In fact, the potential of **L** loaded with Ca(II) should be explored for its ion exchanger properties for removing cations from water.

## 3. Materials and Methods

### 3.1. Chemicals

For the synthetic work, reagents purchased from Aldrich were acetaldehyde, 99 %; bromochloromethane, 99 %; 18-crown-6, 99 %; 99 %; ethylamine; ethyl bromoacetate, 98 %; formaldehyde, (37 % in water); resorcinol, 98 % while those from Fisher were phosphorous pentoxide, P_4_O_10;anhydrous_ potassium carbonate; potassium chloride; hydrochloric acid, 37%; acetone, HPLC grade; acetonitrile (MeCN), HPLC grade; dichloromethane (DCM), HPLC grade; ethanol (EtOH), absolute; and dimethyl sulfoxide (DMSO). *N,N*-diethyl chloroacetamide, 98 % was purchased from Acros Organics. Solvents used in NMR measurements were acetone [(CD_3_)_2_CO]; acetonitrile (CD_3_CN); chloroform (CDCl_3_); methanol (CD_3_OD); and dimethyl sulfoxide (C_2_D_6_SO) purchased from Aldrich. Acetonitrile was placed over calcium hydride in a round bottom flask fitted with a condenser and guarded by a drying tube containing calcium chloride [35]. The mixture was refluxed under nitrogen and distilled. The middle fraction of the distilled solvent was used directly. Methanol HPLC grade was used without further purification. Dimethyl sulfoxide was mixed with calcium hydride and left overnight; it was then filtered. The filtrate was mixed with calcium hydride and fractionally distilled under reduced pressure. The middle fraction of the distilled solvent was used. 

For complexation studies, metal cation salts were purchased from Aldrich: lithium perchlorate (LiClO_4_); sodium perchlorate monohydrate (NaClO_4_.H_2_O), 98 %; potassium perchlorate (KClO_4_), 99 %; rubidium perchlorate anhydrous (RbClO_4_), 99.5 %; magnesium perchlorate hexahydrate (Mg(ClO_4_)_2_.6H_2_O), 99 %; calcium perchlorate tetrahydrate (Ca(ClO_4_)_2_.4H_2_O), 99 %; strontium perchlorate hydrate (Sr(ClO_4_)_2_.H_2_O); barium perchlorate hydrate (Ba(ClO_4_)_2_.H_2_O), 98 %; lead(II) perchlorate trihydrate (Pb(ClO_4_)_2_.3H_2_O), 98 %; cadmium perchlorate hydrate (Cd(ClO_4_)_2_.H_2_O); zinc perchlorate hexahydrate (Zn(ClO_4_)_2_.6H_2_O); silver perchlorate (AgClO_4_), 99.9 %; and mercury (II) perchlorate hydrate (Hg(ClO_4_)_2_.H_2_O), 99.998 %. All salts were dried over phosphorous pentoxide under vacuum for several days before use. Salts were checked by ^1^H NMR to ensure the removal of water. The absence of the water peak, other than the one already in the solvent, in the spectrum indicated the dryness of the salt.

### 3.2. Preparation of 5,11,17,23-Tetra-Tert-Butylcalix[4]arene-25, 26, 27, 28-Tetrol; ***1***

**Scheme 1:**
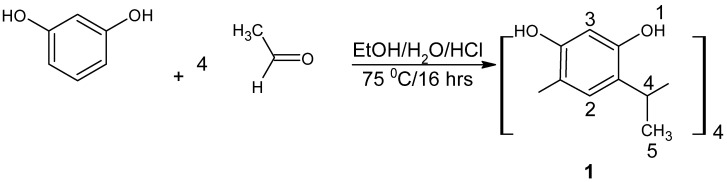
Procedure used for the synthesis of **1**.

The synthetic procedure for the preparation of calix[4]resorcinarene, **1**, is shown in Scheme 1. 

Resorcinol (34 g, 309 mmol) was dissolved in an ethanol–water (1:1) solvent mixture. The mixture was cooled down to 20 °C. Then HCl (30 cm^3^) was gradually poured into the solution to provide the acidic medium, and acetaldehyde (17.4 cm^3^, 311 mmol) was added very slowly to the solution. The solution was left at room temperature and then refluxed at 75°C. The reaction was left for 16 h. The solid was separated by filtration. The precipitate was washed with an ethanol–water (1:1) solvent mixture. The solid was then recrystallized from acetonitrile to give calix[4]resorcinarene (80 % yield). The compound was characterized by ^1^H-NMR in d_6_-DMSO at 298 K and microanalysis. ^1^H-NMR, (300 MHz, in d_6_-DMSO); (ppm); δ=8.52 (s, 2H, H-1), 6.77 (s, 1H, H-3), 6.14 (s, 1H, H-2), 4.45(q, 1H, H-4), 1.301 (d, 3H, H-5). Microanalysis was carried out at the University of Surrey. Calculated %: C, 70.57; H, 5.92. Found %: C, 70.73; H, 5.83

### 3.3. Synthesis of 4,6,10,12,16,18,22,24 Diethyl Amino Carbonyl Methoxy Calix[4]resorcinarene, ***L***

**Scheme 2:**
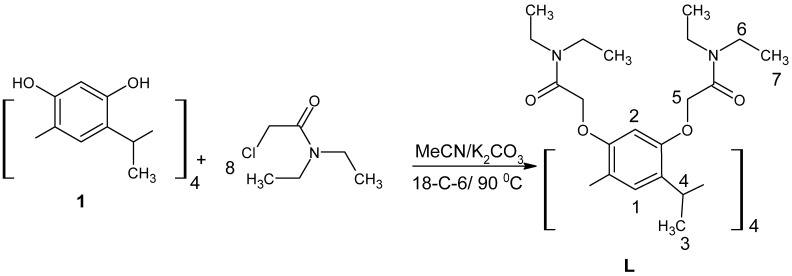
Procedure used for the synthesis of **L**.

The synthetic procedure for the preparation of the calix[4]resorcinarene amide, **L**, is shown in Scheme 2.

Calix[4]resorcinarene, **1** (2.275 g, 4.17 mmol) was dissolved in acetonitrile, anhydrous, K_2_CO_3_ (9.22 g, 66.72 mmol) and 18-crown-6 (2.204 g, 8.34 mmol) was added to the solution and refluxed at 90 °C. Reaction was followed by TLC (eluent DCM:MeOH:ethyl acetate 9.5:1.5:0.5) then *N,N* diethyl chloroacetamide (10 g, 66.8 mmol) was carefully dropped into the solution and left overnight. Acetonitrile was removed using a vacuum rota evaporator. Then the solid was dissolved in DCM and extracted with an aqueous solution of sodium bicarbonate. The organic phase was dried off, the solvent and the oily residue was treated with hexane and filtered. The product was recrystallized from acetonitrile. The solid was dried in a pistol dryer (40% yield). The compound was characterised by ^1^H NMR in CD_3_CN at 298 K and microanalysis, ^1^H NMR spectrum is provided in the Supplementary Information Figures S1–S12. ^1^H NMR, (300 MHz, in CD_3_CN); δ (ppm); δ = 6.73 (s, 1H, H-2), 6.34 (s, 1H, H-1), 4.70 (q, 1H, H-4), 4.50 (s, 2H, H-5), 3.31 (t, 3H, H-7), 1.42 (d, 3H, H-3), 1.10 (q, 2H, H-6). Microanalysis was carried out at the University of Surrey. C_80_H_136_N_8_O_16_ Calculated %: C, 66.27; H, 8.34; Found %: C, 66.59; H, 8.19.

### 3.4. Nuclear Magnetic Resonance Measurements

#### ^1^H NMR Measurements

A Bruker AC-300 (Bruker BioSpin GmbH, Rheinstetten, Germany) pulsed Fourier Transform NMR spectrometer of special frequency 300.135 MHz, was used for recording ^1^H NMR measurements at 298 K. Tetramethyl silane was used as an internal standard. Typical operating conditions for routine proton measurements involved pulse or flip angle of 30°, spectra width (SW) of 20.7 ppm, spectral frequency (SF) of 300.137 MHz, delay time of 1.60 s, Acquisition time (AQ) of 1.819 s, and line broadening of 0.55 Hz. Spectra were processed using TopSpin 3.6.2 software. 

### 3.5. Conductometric Measurements

Conductivity measurements were carried out using the Wayne-Kerr B642 Auto balance Universal Bridge as described elsewhere [36]. In order to determine the stoichiometry of the metal ion complex, conductometric titrations were carried out in the appropriate solvent. To perform these measurements, a solution of the metal–ion salt in an appropriate solvent (25 cm^3^) was placed in the cell. Then the electrodes were inserted into the cell. The solution was continuously stirred during the course of the titration. The vessel was sealed and placed in a thermostatic bath at 298.15 K for 20 min to reach thermal equilibrium. The titrant (ligand ≈ 5 × 10^−3^ mol.dm^−3^) was added using a hypodermic syringe. The conductance S (Ω^−1^) was measured after each addition once equilibrium was achieved. The plots of molar conductance, Λm (Ω^−1^.cm^2^.mol^−1^), against the ligand–cation ratio ([L]/[M^+^]) were drawn.

### 3.6. Titration Calorimetric Measurements

Calorimetric titrations were carried out in the Tronac 450 calorimeter [37,38] and the Thermal Activity Monitor TAM 2277 microcalorimeter (LKB Products, Stockholm, Sweden) as described elsewhere [39,40]. 

### 3.7. Potentiometric Titrations for the Determination of Stability Constants

The stability constant of **L** with the silver cation in methanol was determined at 298.15 K. Thus, the ligand solution (5.4 × 10^−3^ mol.dm^−3^) was prepared in a solution of TBAP (0.05 mol.dm^−3^). The working electrode was placed in a solution of silver perchlorate (1 × 10^−3^ mol.dm^−3^). The solution was titrated with the ligand solution prepared in TBAP solution [41]. Potential readings were taken after each addition and were used for the calculation of the stability constant of this system in the appropriate solvent. 

### 3.8. Molecular Simulation Studies 

Argus Lab 4.0.1 was the software used for molecular simulation studies. Every structure was ready for analysis by optimizing the H-bond. Molecular mechanics (MM) method was selected where the universal force field (UFF) approach was used [42,43]. For MD studies of **L** in methanol and acetonitrile, the simulations were conducted in a grid box of 21.729 × 17.7298 × 12.018 Å for methanol and 21.875 × 18.28 × 21.171 Å for acetonitrile. For the number of solvent molecules, 20 were introduced to represent the first solvation shell. The interactions between **L** and calcium, mercury, and lead cations were investigated, and the binding energy in kJ.mol^−1^ was computed. 

### 3.9. Calcium Uptake from Aqueous Solution by Calix[4]resorcinarene Amide, ***L***

**L** extracting ability to remove calcium ion salt from aqueous solutions was conducted through batch experiments in which the receptor in the solid state was equilibrated with aqueous solutions containing the relevant cation salt at the standard temperature of 298 K. The Flame Photometer (Model 360) was used to analyze the initial and the equilibrium concentrations of cationic species (as perchlorate salt) in aqueous solution. The optimum conditions for extraction were determined as follows.

### 3.10. Optimum Mass of ***L*** for the Removal of Calcium from Aqueous Solutions

The mass effect was investigated by adding different quantities (0.005–0.150 g) of the material used for extraction to a set of nine test tubes, each one containing an aqueous solution of the calcium salt (as perchlorate salt) (10 cm^3^, 1 × 10^−1^ mol.dm^−3^). The test tubes were then shaken on a rota-mixer for 5 min to ensure homogenous mixing and left overnight in a thermostat water bath at 298 K. After equilibrium was attained, aliquots of the solution were taken and analyzed, after which the percentage of extraction (%E) was calculated using Equation (10).

%E = (ci − ceq)/ci × 100(10)

In Equation (10), ci and cf denote the initial and the calcium equilibrium concentrations (mmol.dm^−3^) in water.

### 3.11. The Effect of pH Solution of the Aqueous Solution on the Extraction Process

The pH effect on the extraction of the calcium salt solution in aqueous medium by **L** was examined in the 3–10.5 pH range. The initial concentration of the cation (1 × 10^−1^ mmol.dm^−3^) was kept constant in de-ionized water at the same time as the solution pH was adjusted to the desired values by the addition of 0.1 mol.dm^−3^ sulfuric acid or 0.1 mol.dm^−3^ sodium hydroxide using a digital pH metre equipped with a combined pH electrode. Samples were then left overnight at 298 K. Afterwards, the supernatants were analyzed for calcium residual concentration by as described above and the %E was calculated using Equation (10). The obtained optimal pH was used for further experiments.

### 3.12. Uptake Capacity of ***L*** for Calcium Ion

The uptake capacity of the material for the removal of calcium species from aqueous medium was determined under static conditions by the batch equilibrium technique. Experiments were carried out at 298 K by varying the cationic species concentration (1 × 10^−3^–1 × 10^−1^ mol.dm^−3^) under study while fixing the mass (optimum mass) of the material used for extraction. The mixtures were shaken by the rota-mixer for 5 min and then left overnight to reach equilibrium. The tubes were then placed in a water bath and the temperature was maintained at 298 K. After equilibrium was attained (no further changes were observed in the concentration of the calcium salt in contact with the material), the cation was analyzed using the Flame Photometer. The amount of the calcium removed per gram of the solid material was calculated from the difference in their initial and equilibrium concentrations in the aqueous solutions.

(11) $$Q_{eq}=\frac{\left(Ci-Ceq\right)\times V}{m}$$

In Equation (11), Q_eq_ is the amount of cation taken up by **L** material (mmol/g), V is the volume of the sample (dm^3^), ci and ceq are the initial and the equilibrium calcium concentrations (mmol.dm^−3^), and m (g) is the amount of the solid material used. 

### 3.13. Kinetics of Calcium Extraction Processes from Aqueous Solutions by ***L***

The kinetics of the extraction process were examined under the same batch conditions described above, but at different time intervals (between 30 and 1440 min). The removal uptake (mmol.g^−1^) was calculated; half-life values for Ca(II) removal were determined from the plots of the material uptake vs. the time in minutes. The following equation for pseudo first order model was applied.

(12) $$\log\;(\mathrm{Qe}-\mathrm{Qt})=\;\mathrm{logQe}-\frac{k1}{2.303}t$$

Qe is the uptake removal in mmol g^−1^, Qt (mmol.g^−1^) the amount of removal at time (min), and k_1_ the Lagergren rate constant of calcium ion removal (min^−1^).

### 3.14. Effect of Interfering Cations on the Uptake of Calcium by ***L***

To investigate if there is any competition between the different cations to be extracted by **L**, multiple cation solutions containing lead (Pb(II)), cadmium (Cd(II)), barium (Ba(II)), and strontium (Sr(II)) as perchlorate salts were prepared. All cations were present at equimolar concentrations (1 × 10^−1^ mol.dm^−3^). The removal ability of the material was tested using a batch procedure. The remaining concentration of the calcium was analyzed.

## 4. Conclusions

From the above discussion the following conclusions are drawn:

(i) In searching for applications, the thermodynamics characterization of the complexation process provides a quantitative assessment of the selectivity of the ligand for a given cation relative to other. These studies are essential particularly in extraction processes where the issue of selectivity plays a major role in the selection of a receptor to target a particular ion or neutral species in a given medium. 

(ii) Although ^1^H NMR studies on the L receptor are indicative that unlike in methanol, acetonitrile must sit in the cavity of the receptor as suggested by molecular simulation studies, there is no indication that this is the case for the metal–ion complexes. 

(iii) The complex interplay between ligand-binding energy and cation desolvation is clearly demonstrated in acetonitrile. The weak cation–ligand interaction in methanol is due to the higher solvation of ions in methanol relative to acetonitrile but also the solvation of the ligand in this solvent. There is a competition of the ligand and the solvent for the cation. In MeOH, the latter is stronger and therefore weaker complexes are formed. In addition, the affinity of the solvent for the donor atoms of the ligand through hydrogen formation reduces even further and, in some cases, impedes complex formation in methanol.

(iv) The remarkable high capacity of L to remove Ca(II) from water even in the presence of interfering ions makes this receptor suitable for commercialisation purposes.

Considering water with a typical hardness of 200 mg/L of Ca(II), the capacity of L would be sufficient to treat approximately 24 L of hard water before requiring regeneration.

## Figures and Tables

**Table 1:** ^1^H NMR data for **L** in deuterated acetonitrile and methanol at 298 K (CD_3_OD as reference solvent).

Proton #	CD_3_CN δ (ppm)	CD_3_OD δ (ppm)	Δδ (ppm) (CD_3_CN-CD_3_OD)
H-1	6.34	--	--
H-2	6.73	6.47	0.26
H-3	1.42	1.44	−0.02
H-4	4.70	4.70	0.00
H-5	4.50	4.55	−0.05
H-6	1.10	1.14	−0.04
H-7	3.31	3.39	−0.08
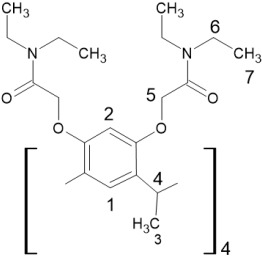 **L**			

**Table 2:** ^1^H NMR chemical shift changes (∆δ, ppm) obtained as the result of metal cation salts’ addition to **L** relative to the free ligand in deuterated CD_3_CN and CD_3_OD at 298 K.

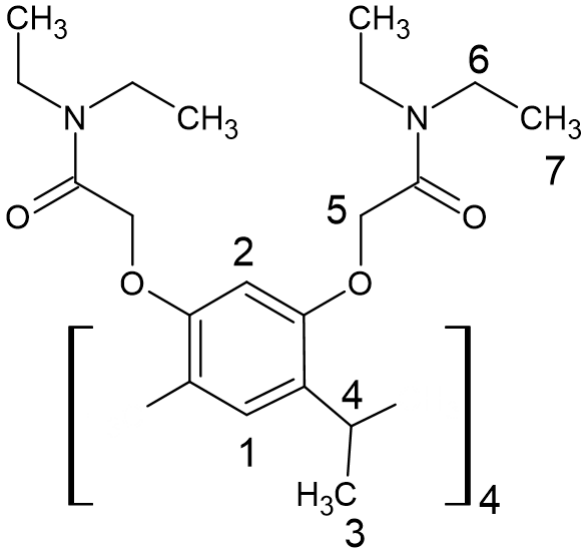 L							
.	**H-1**	**H-2**	**H-3**	**H-4**	**H-5**	**H-6**	**H-7**
Solvent: CD_3_CN							
*δ*_Ref_	6.34	6.73	1.42	4.70	4.50	1.10	3.31
Li(I)	0.16	−0.05	0.12	0.01	−0.02	0.05	0.00
Na(I)	0.16	---	0.05	0.06	0.13	0.01	−0.02
K(I)	−0.10	−0.23	0.02	0.05	0.14	0.00	−0.01
Rb(I)	−0.12	−0.23	0.02	0.05	0.17	0.01	−0.02
Mg(II)	0.16	−0.11	0.08	0.17	−0.03	0.04	0.08
Ca(II)	0.16	−0.17	0.03	0.15	−0.04	0.07	0.07
Sr(II)	0.16	0.08	0.02	0.14	−0.03	0.06	0.03
Ba(II)	0.16	−0.05	0.06	−0.10	---	0.07	−0.08
Zn(II)	0.16	−0.16	0.27	0.14	0.26	0.05	−0.03
Pb(II)	0.16	−0.14	0.12	0.00	0.13	0.05	0.01
Cd(II)	0.16	−0.16	0.27	0.14	0.26	0.05	−0.03
Hg(II)	0.16	−0.10	0.08	0.14	−0.02	−0.01	−0.02
Solvent: CD_3_OD							
*δ*_Ref_	----	6.47	1.44	4.75	4.55	1.14	3.39
Ca(II)	----	0.22	0.07	−0.02	0.04	0.02	0.05
Sr(II)	----	0.23	0.07	0.00	0.05	0.03	0.06
Ba(II)	----	0.17	0.03	−0.02	0.03	0.01	0.01
Ag(I)	----	−0.17	0.03	0.01	0.10	0.05	0.06
Pb(II)	----	----	0.11	−0.10	----	0.07	0.04

**Table 3:** Thermodynamic parameters of complexation of **L** with metal cations in MeCN and MeOH at 298.15 K.

Cation (L:M) (1:1)	Log K_s_	Δ*_c_*G^0^ (kJ.mol^−1^)	Δ*_c_*H^0^ (kJ.mol^−1^)	Δ*_c_*S^0^ (J.K^−1^.mol^−1^)
Solvent: MeCN				
Na(I)	2.5 ± 0.1 ^a^	−14.3 ± 0.3	−70.7 ± 0.5 ^a^	−190
Mg(II)	2.34 ± 0.02 ^a^	−13.4 ± 0.1	−24.2 ± 0.4 ^a^	−36
Ca(II)	6.92 ± 0.02 ^b^	−39.51 ± 0.02	−126.2 ± 0.3 ^b^	−291
Sr(II)	5.58 ± 0.09 ^a^	−31.9 ± 0.5	−31.6 ± 0.2 ^a^	1
Ba(II)	5.64 ± 0.03 ^a^	−32.2 ± 0.2	−29.9 ± 0.6 ^a^	8
Cd(II)	4.0 ± 0.1 ^a^	−22.8 ± 0.5	−29.3 ± 0.1 ^a^	−22
Zn(II)	1.89 ± 0.01 ^a^	−10.79 ± 0.03	−16.41 ± 0.02 ^a^	−19
Hg(II)	3.2 ± 0.1 ^a^	−18.37 ± 0.01	−85.1 ± 0.1 ^a^	−224
Pb(II)	6.3 ± 0.1 ^b^	−35.8 ± 0.3	−99.9 ± 0.3 ^b^	−215
Solvent: MeOH				
Ca(II)	4.0 ± 0.2 ^a^	−22.9 ± 0.2	−1.9 ± 0.5	71
Ba(II)	0.8 ± 0.1 ^a^	−4.7 ± 0.4	−62.9 ± 0.3	−195
Pb(II)	1.5 ± 0.1 ^a^	−8.5 ± 0.3	−136.4 ± 0.4	−429

^a^ Direct calorimetry. ^b^ Competitive calorimetry.

## Data Availability

The data are presented in the article/Supplementary Information. Any inquiry can be directed to the corresponding author.

## Supplementary Materials

The following supporting information can be downloaded at: https://www.mdpi.com/article/10.3390/ijms26168043/s1.
