# Supplementary material for: Calix[4]resorcinarene Amide Derivative: Thermodynamics of Cation Complexation Processes and Its Remarkable Properties for the Removal of Calcium (II) from Water
Source: Int J Mol Sci. 2025 Aug 20;26(16):8043. doi: 10.3390/ijms26168043 (PMC12386828; doi:10.3390/ijms26168043)
Supplement: Supplementary file 1 [file ijms-26-08043-s001.zip › ijms-3621196-supplementary.pdf]

Supplementary Information

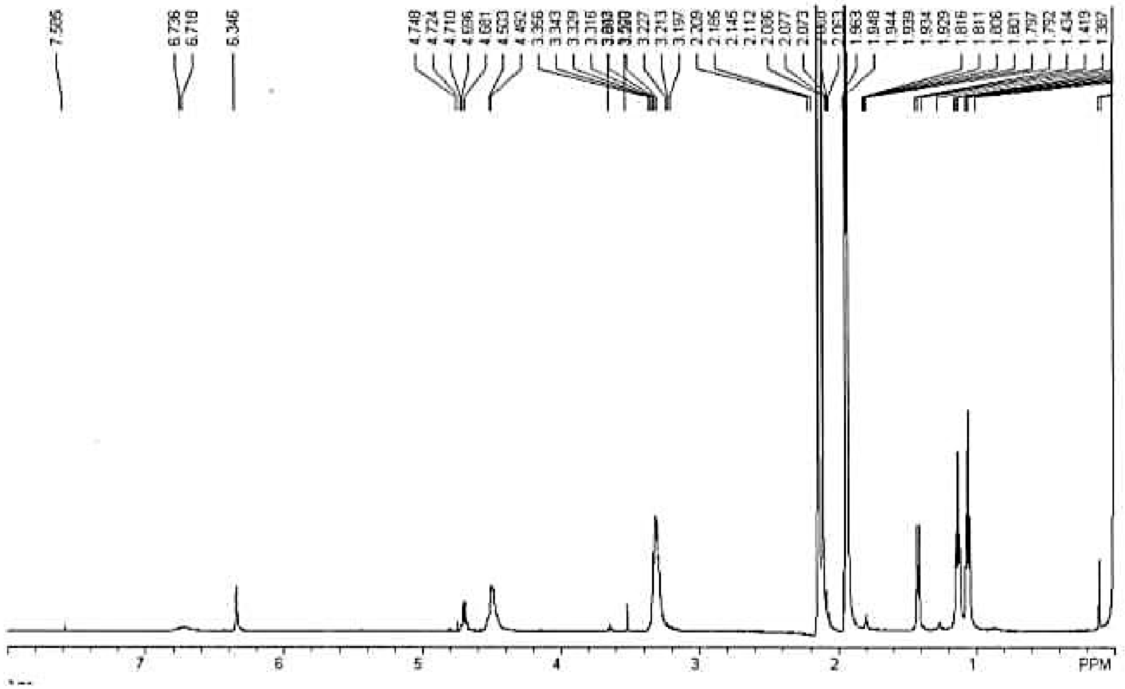

Figure S1 ^{1}H NMR of L in CD_3CN at 298 K

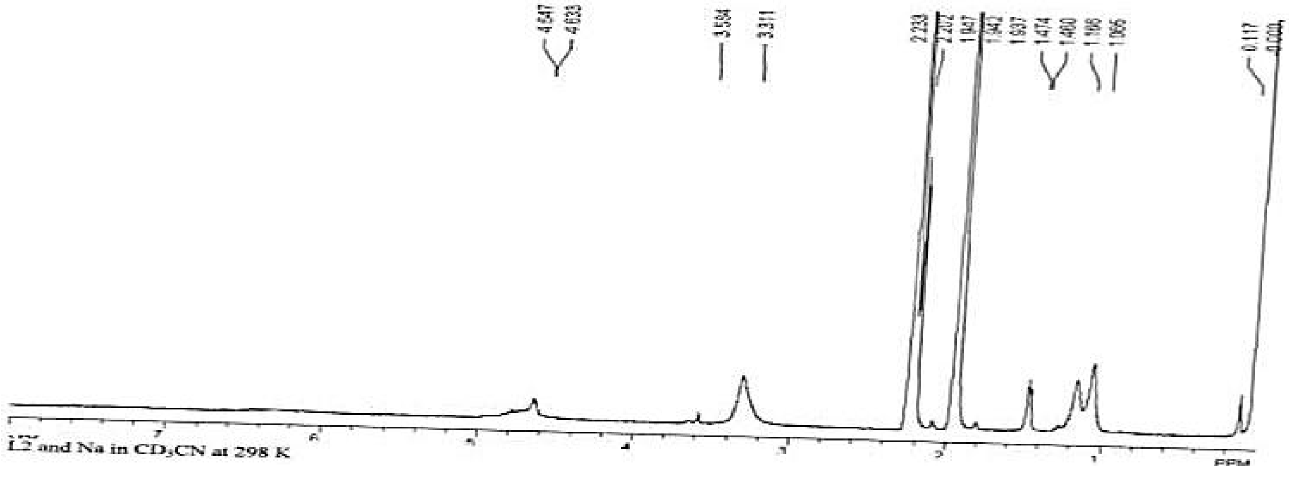

Figure S2 ^{1}H NMR of L and Na(I) in CD_3CN at 298K

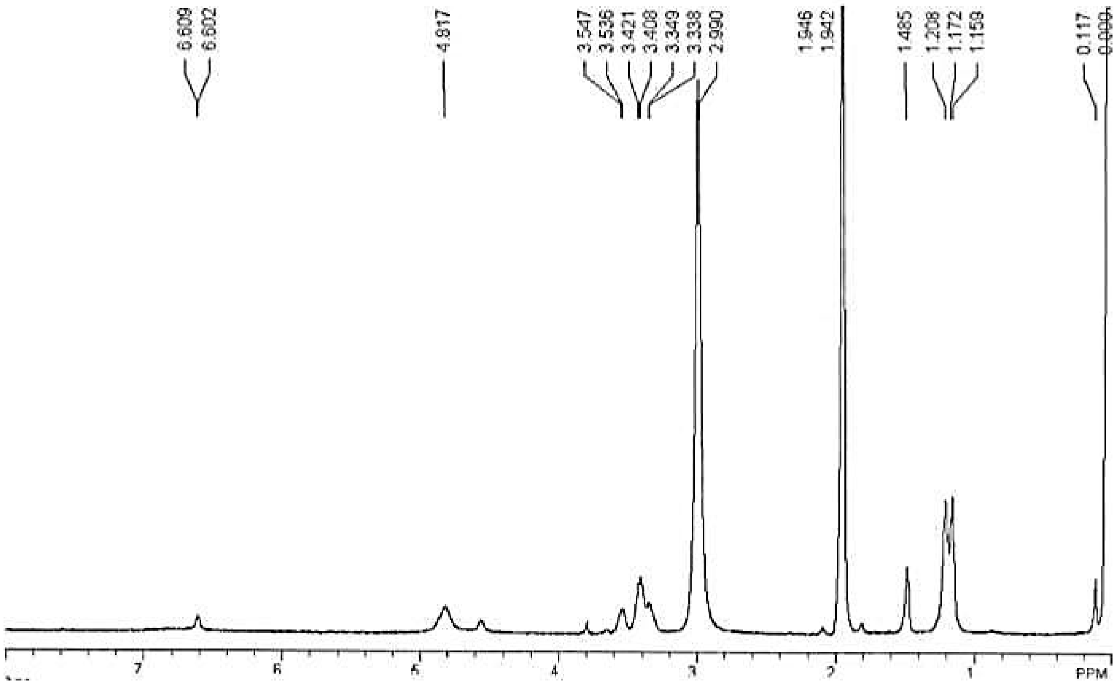

Figure S3 ^{1}H NMR of L and Ca(II) in CD_3CN at 298 K

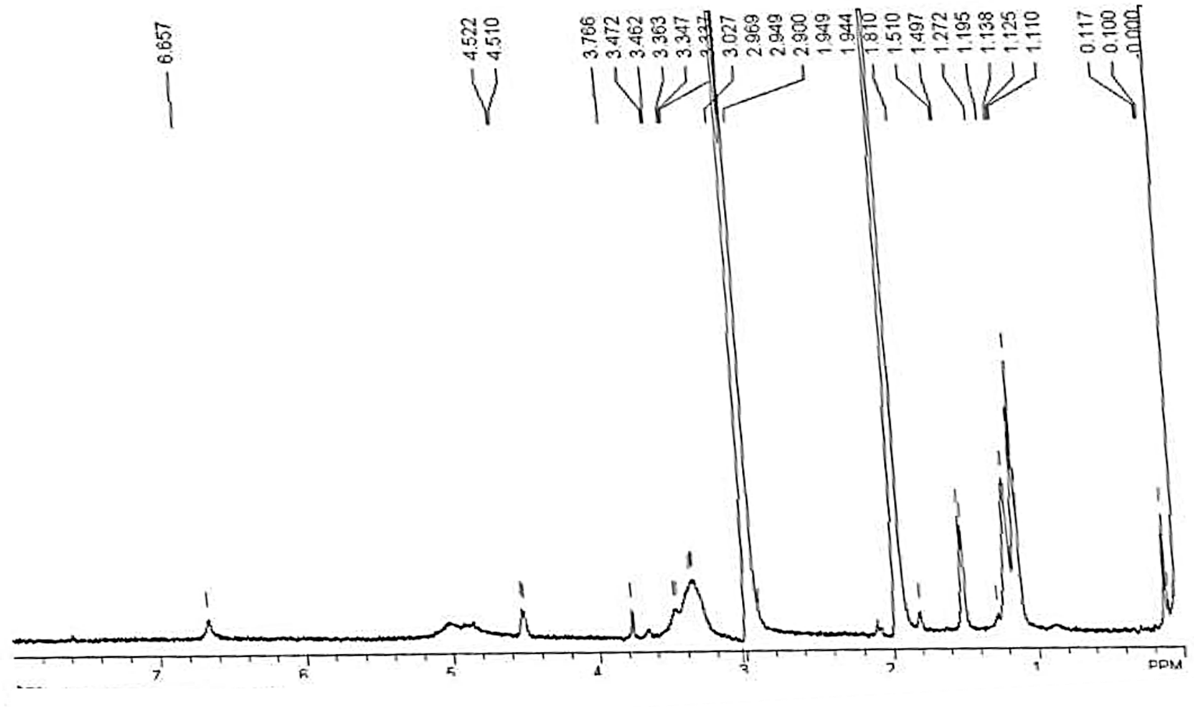

Figure S4 ^{1}H NMR of L and Sr(II) in CD_3CN at 298K

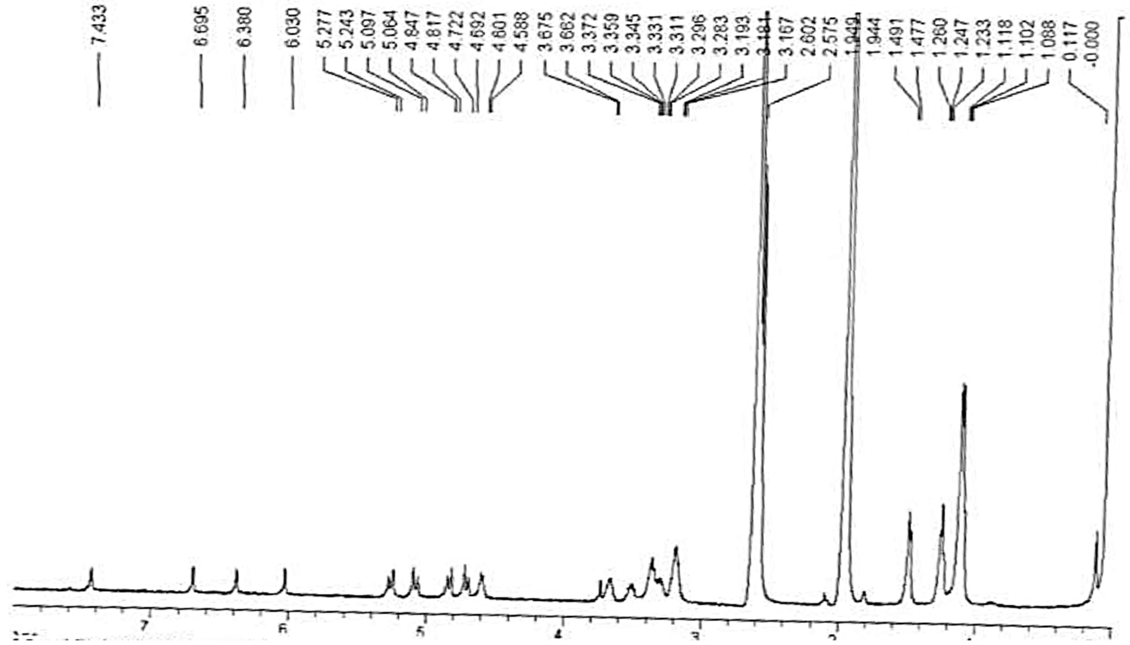

Figure S5 ^{1}H NMR of L and Ba(II) in CD_3CN at 298K

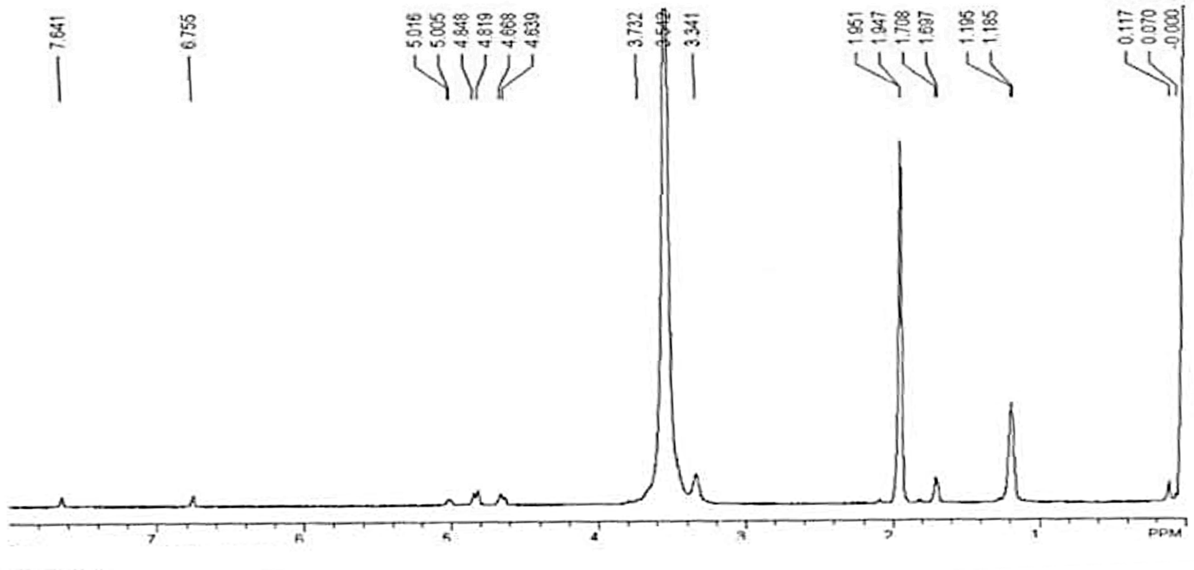

Figure S6 ¹H NMR of L and Zn(II) in CD_3CN at 298 K

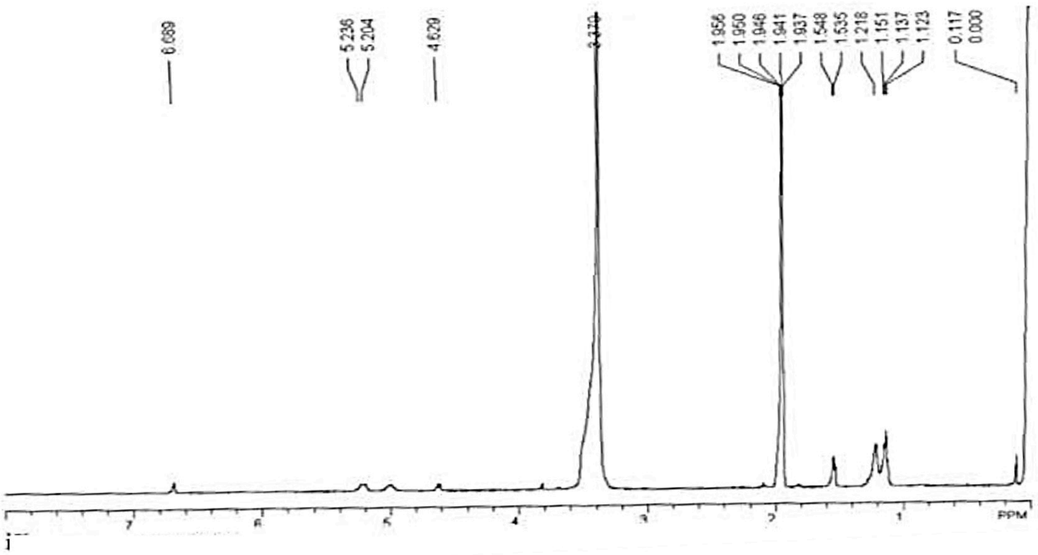

Figure S7 ¹H NMR of L and Pb(II) in CD_3CN at 298 K

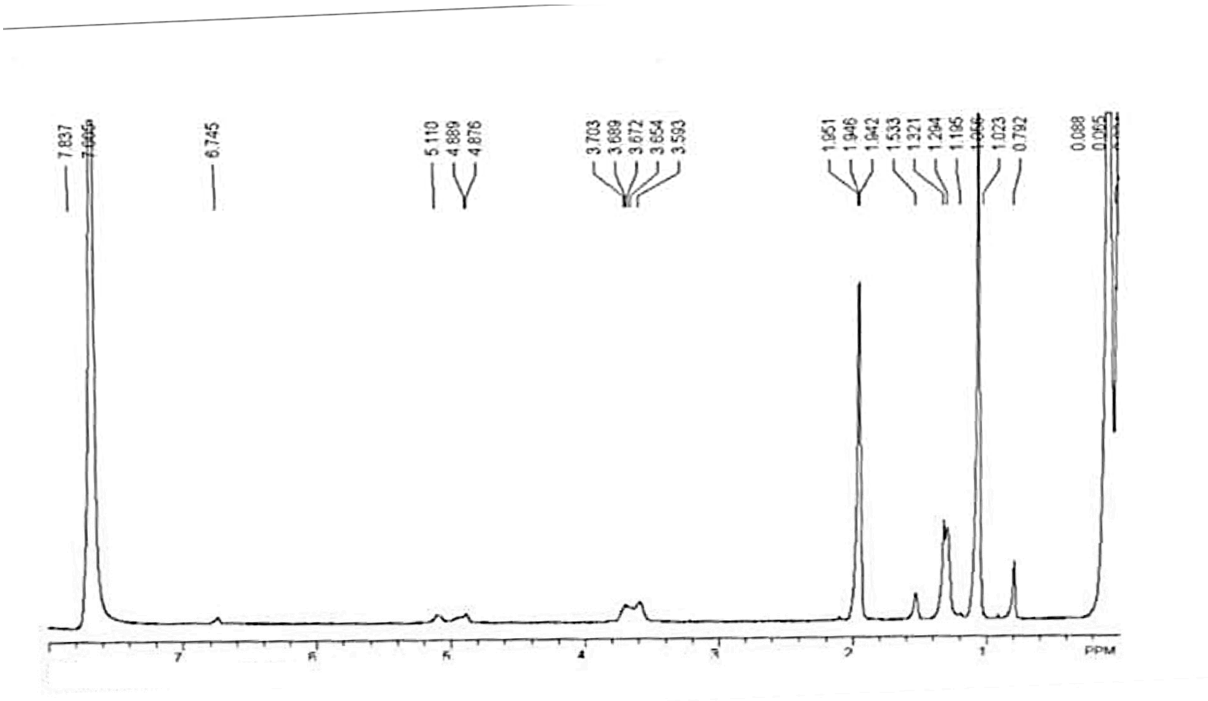

Figure S8 ^{1}H NMR of L and Hg(II) in CD_3CN at 298K

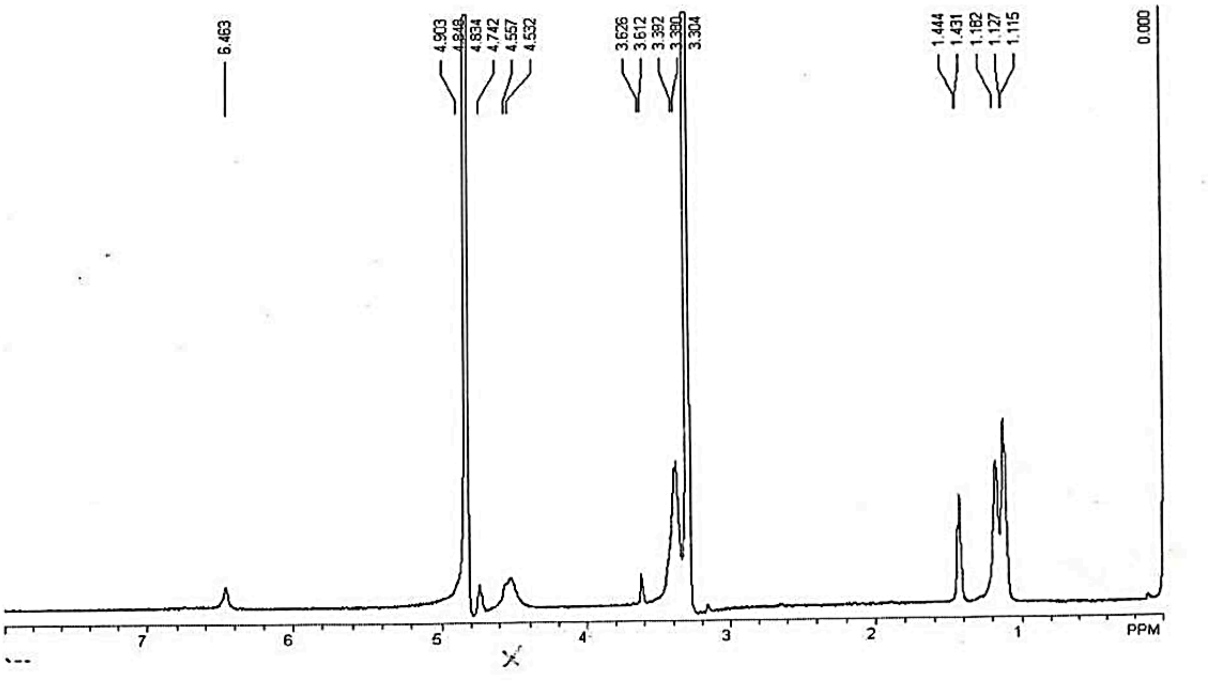

Figure S9 ^{1}H NMR of L in CD_3OD at 298 K

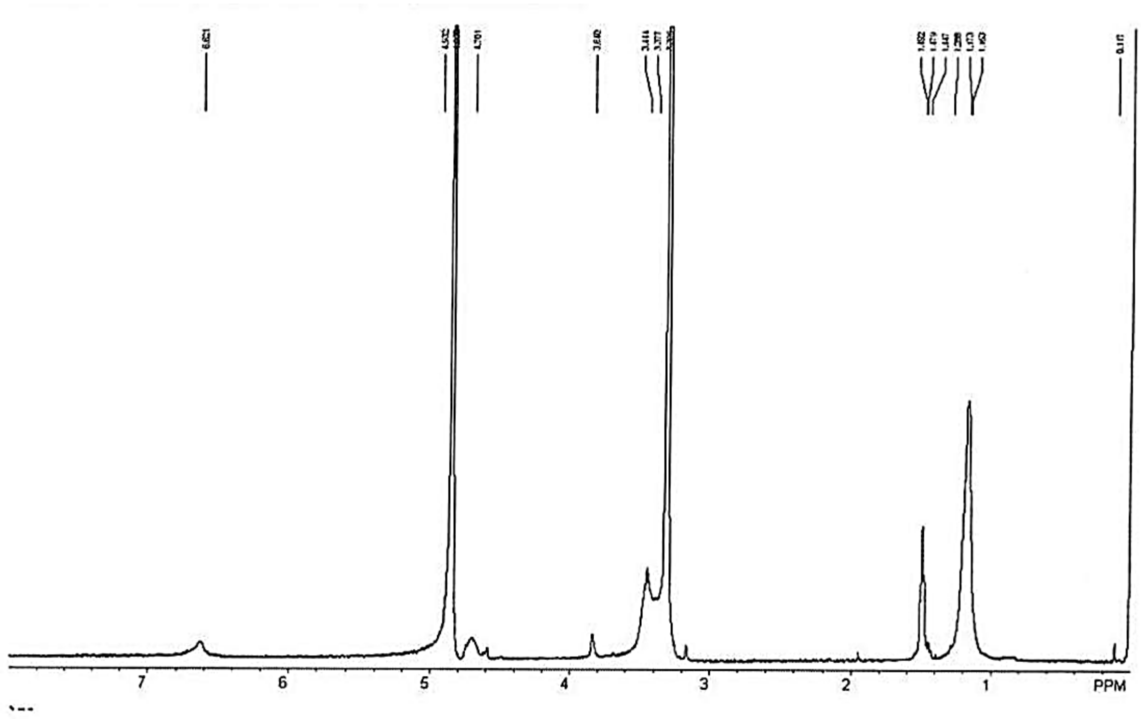

Figure S10 ^{1}H NMR of L with Ca(II) in CD_3OD at 298 K

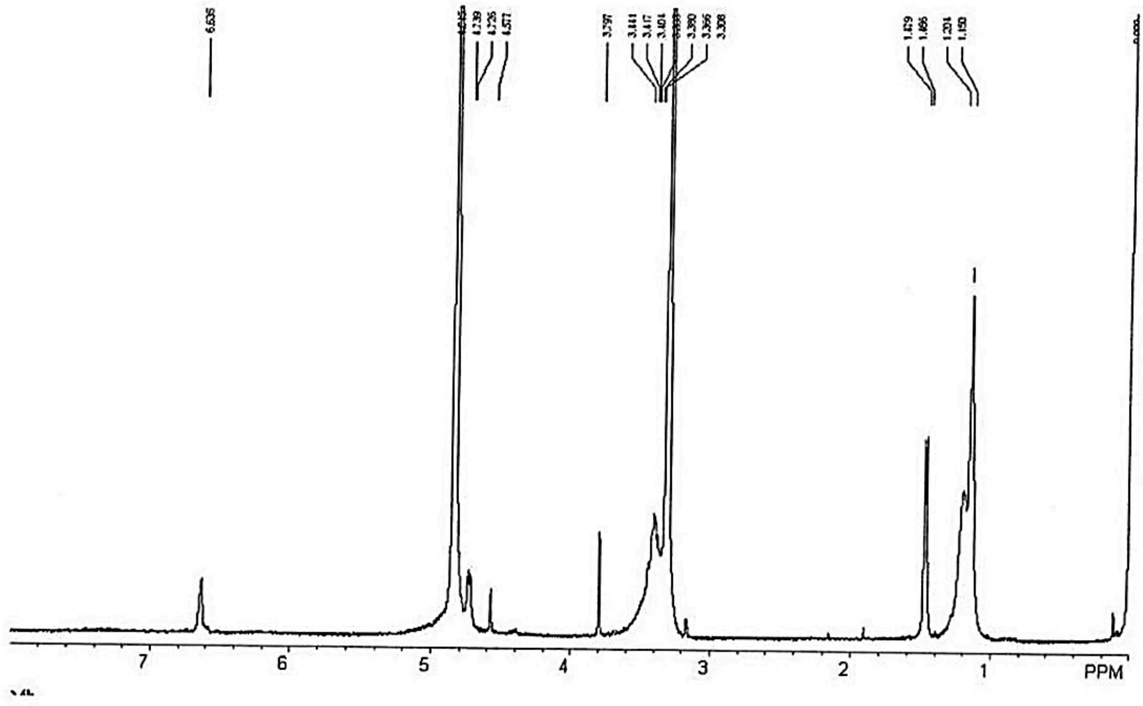

Figure S11 ^{1}H NMR of L with Ba(II) in CD_3OD at 298 K

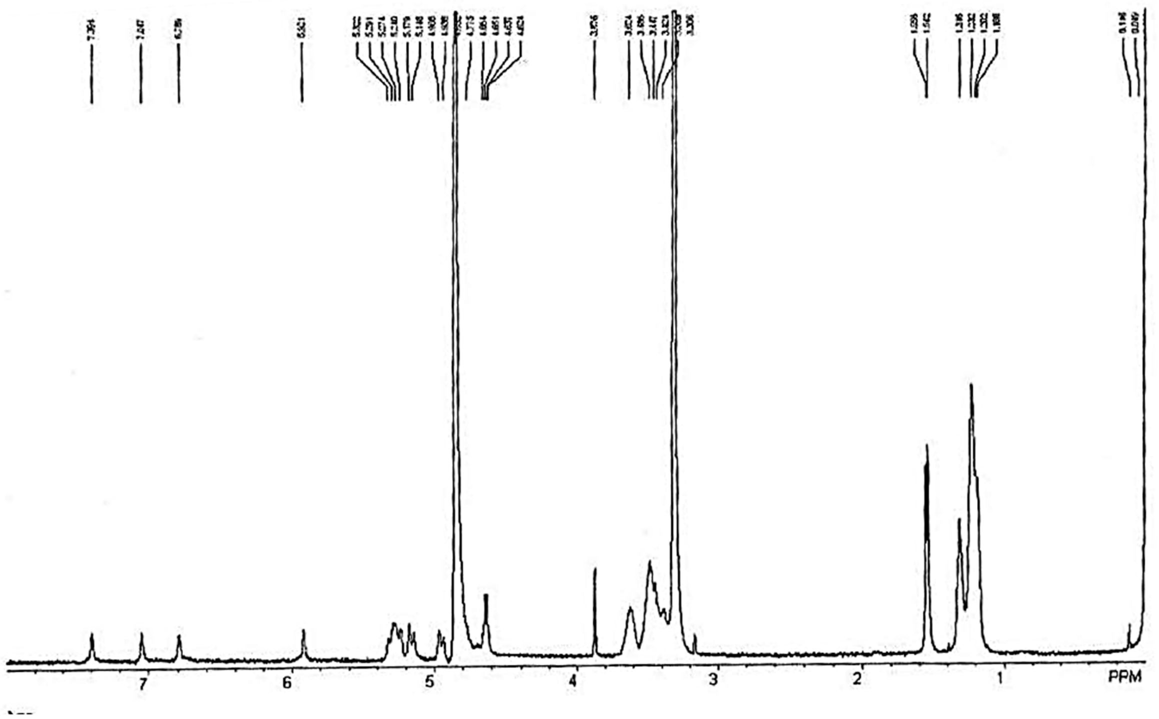

Figure S12 ^{1}H NMR of L with Pb(II) in CD_3OD at 298 K
